# Laser Fiber Displacement Velocity during Tm-Fiber and Ho:YAG Laser Lithotripsy: Introducing the Concept of Optimal Displacement Velocity

**DOI:** 10.3390/jcm11010181

**Published:** 2021-12-29

**Authors:** Frederic Panthier, Thibault Germain, Cyril Gorny, Laurent Berthe, Steeve Doizi, Olivier Traxer

**Affiliations:** 1 GRC n°20, Groupe de Recherche Clinique sur la Lithiase Urinaire, Hôpital Tenon, Sorbonne Université, 75020 Paris, France; frederic.panthier@aphp.fr (F.P.); thibgermain@msn.com (T.G.); steeve.doizi@aphp.fr (S.D.); 2 Service d’Urologie, Assistance-Publique Hôpitaux de Paris, Hôpital Tenon, Sorbonne Université, 4 Rue de la Chine, 75020 Paris, France; 3 PIMM, UMR 8006 CNRS-Arts et Métiers ParisTech, 151 bd de l’Hôpital, 75013 Paris, France; cyril.gorny@ensam.eu (C.G.); laurent.berthe@cnrs.fr (L.B.); 4 Service d’Urologie, Assistance-Publique Hôpitaux de Paris, Hôpital Européen George Pompidou, 20 Rue Leblanc, 75015 Paris, France

**Keywords:** thulium fiber laser, Ho:YAG laser, ablation volumes, urolithiasis, lithotripsy, endourology, in vitro

## Abstract

Background: Endocorporeal laser lithotripsy (EL) during flexible ureteroscopy (URS-f) often uses “dusting” settings with “painting” technique. The displacement velocity of the laser fiber (LF) at the stone surface remains unknown and could improve EL’s ablation rates. This in vitro study aimed to define the optimal displacement velocity (ODV) for both holmium:yttrium-aluminium-garnet (Ho:YAG) and thulium fiber laser (Tm-Fiber). Methods: A 50W-TFL (IRE Polus^®^, Moscow, Russia) and a 30W-MH1-Ho:YAG laser (Rocamed^®^, Signes, Provence-Alpes-Côte d’Azur, France), were used with 272 µm-Core-Diameter LF (Sureflex, Boston Scientific^©^, San Jose, CA, USA), comparing three TFL modes, “fine dusting” (FD: 0.05–0.15 J/100–600 Hz); “dusting” (D: 0.5 J/30–60 Hz); “fragmentation” (Fr: 1 J/15–30 Hz) and two Ho:YAG modes (D: 0.5 J/20 Hz, Fr: 1 J/15 Hz). An experimental setup consisting of immerged cubes of calcium oxalate monohydrate (COM) stone phantoms (Begostone Plus, Bego^©^, Lincoln, RI, USA) was used with a 2 s’ laser operation time. LF were in contact with the stones, static or with a displacement of 5, 10 or 20 mm. Experiments were repeated four times. Stones were dried and µ-scanned. Ablation volumes (mm^3^) were measured by 3D-segmentation. Results: ODV was higher in dusting compared to fragmentation mode during Ho:YAG lithotripsy (10 mm/s vs. 5 mm/s, respectively). With Tm-Fiber, dusting and fragmentation OVDs were similar (5 mm/s). Tm-Fiber ODV was lower than Ho:YAGs in dusting settings (5 mm/s vs. 10 mm/s, respectively). Without LF displacement, ablation volumes were at least two-fold higher with Tm-Fiber compared to Ho:YAG. Despite the LF-DV, we report a 1.5 to 5-fold higher ablation volume with Tm-Fiber compared to Ho:YAG. Conclusions: In dusting mode, the ODV^Tm-Fiber^ is lower compared to ODV^Ho:YAG^, translating to a potential easier Tm-Fiber utilization for “painting” dusting technique. The ODV determinants remain unknown. Dynamic ablation volumes are higher to static ones, regardless of the laser source, settings or LF displacement velocity.

## 1. Introduction

Endocorporeal laser lithotripsy (ELL) is currently the gold-standard for the treatment of renal stones during flexible ureteroscopy (f-URS) [1]. Since its first description in 1992 in the field of urology, the holmium:yttrium-aluminium-garnet (Ho:YAG) pulsed laser has become the reference laser source for ELL, due to its safety profile and efficiency to ablate urinary stones [2]. The recently-authorized thulium fiber laser (Tm-Fiber) has demonstrated promising in vitro results, challenging the leadership of the Ho:YAG [3,4]. If the structural characteristics of Tm-Fiber can explain its superiority in terms of efficiency (mm^3^/s) and efficacy (J/mm^3^), its consecutive bubbles dynamics support also its two-fold higher ablation rates [5,6]. Overcoming this technologic comparison, the efficiency determinants of ELL have not yet been fully demonstrated. If international recommendations recognize two influencing factors: stone size, i.e., maximum diameter and stone density (Hounsfield units), additional determinants are suggested: stone volume, stone location, laser source and laser settings, consumable (laser fiber, ureteroscope, pumping system, ureteral access sheath) and lithotripsy method (dusting technique, pop-dusting and pop-corning) [7]. Considering the dusting technique, the painting method is widely acknowledged, with the one pulse-on locus objective to avoid the burnback effect and the production of significant fragments, and to optimize the ablated stone volume [8]. To date, the stone ablation efficiency depends on the ability and habits of the surgeon to dust stone (production of fine fragments, able to be spontaneously evacuated through the urinary tract), now evaluated by the efficacy (J/mm^3^) and efficiency (mm^3^/s) ratios [9,10,11,12]. No study has ever determined the optimal displacement velocity (ODV) during ELL with both Ho:YAG or Tm-Fiber lasers.

This in vitro study aimed to evaluate the ODV for both Ho:YAG and Tm-Fiber laser within the laser settings. Secondarily, we intended to compare their static and dynamic ablation efficiency, based on ablation volumes.

## 2. Materials and Methods

### 2.1. Superpulsed Thulium Fiber Laser and Holmium:YAG Generators

A 50 W TFL generator (IRE Polus^®^, Moscow, Russia) with a wavelength of 1940 nm was compared to a 30 W Ho:YAG laser (MH1Rocamed^®^, Monaco-Ville, Monaco) with a wavelength of 2120 nm. We connected 272µm Core-Diameter laser fibers (CDF) (Sureflex, Boston Scientific^©^, San Jose, CA, USA). Before experiments, both laser fibers were sectioned by ceramic scissors to assess their real core-diameter under optical microscopy (Zeiss^©^, Oberkochen, Germany). We compared three Tm-Fiber (“Fine dusting” (FD1: 0.05/300 Hz; FD2: 0.15 J/100 Hz), “dusting” (D: 0.5 J/30 Hz) and “fragmentation” (Fr: 1 J/15–30 Hz)) and two Ho:YAG (“dusting” (D: 0.5 J/20 Hz) and “fragmentation” (Fr: 1 J/15 Hz)) lithotripsy modes.

### 2.2. Stone Phantoms

4 cm^3^ pavements of BegoStones were produced according to previously described techniques [13]. We aimed to reproduce calcium oxalate monohydrate (Hard) stones, using a “powder to water” ratio of 15:3. After production, a drying period of 48 h at 30 °C was observed to minimize the heterogeneity. Before laser emission, stone phantoms were immerged into a saline solution for 30 min.

### 2.3. Experimental Setup

**Figure 1:**
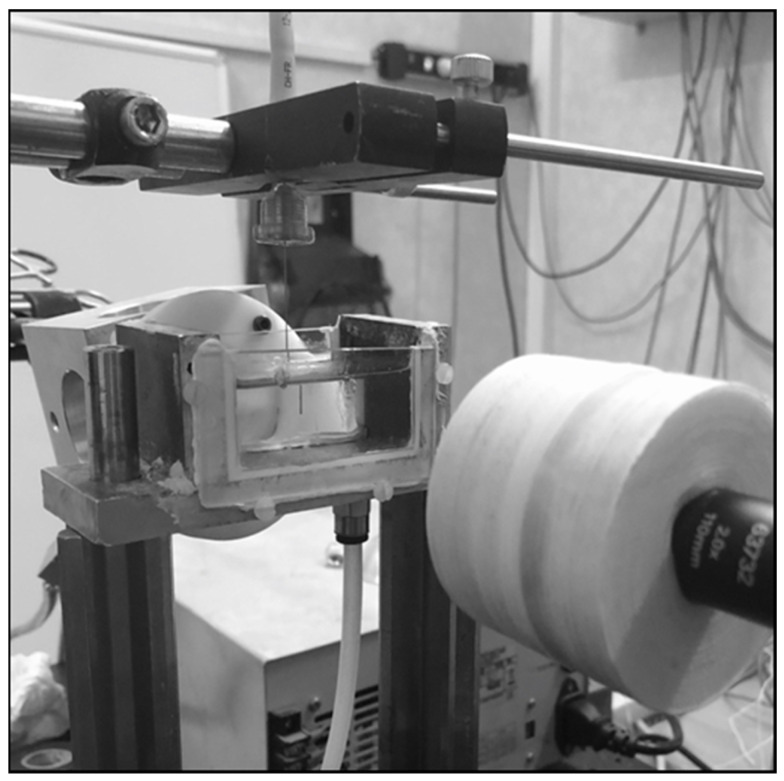
Experimental setup: laser fiber vertically disposed in a cuvette filled with 0.9% saline solution and connected with the six-axes robotic arm.

Stone phantoms were fully immerged with saline solution at ambient temperature and fixed into a bench model. The laser fiber tip was placed perpendicularly and in contact with the surface of the stone. A specific fiber support was manufactured in order to assure the permanent contact with the artificial stone during laser emission (Figure 1). First, a two seconds-firing was executed without displacement, in order to determine the static ablation rate. Then, a robotic six-axes arm (KR6R900, Kuka International^©^, Augsbourg, Germany) was used to realize multiple linear trajectories: 5 mm, 10 mm and 20 mm, maintaining a displacement duration of two seconds. The laser emission and the robotic arm were initiated and stopped jointly by computational command. All experiments were repeated four times. After laser emission, stones were dried as described above.

**Figure 2:**
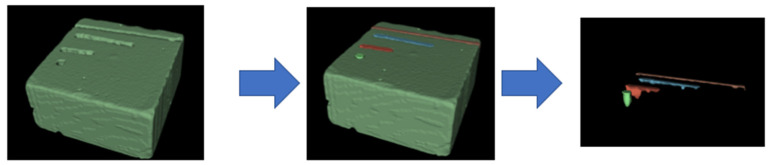
Segmentation method to determine the ablated volume, using 3DSlicer; defining the «stone segment»; defining the «air segments»; subtractive logical operations and splitting to obtain distinct ablated volumes.

To assess the ablation volumes, a three-dimensional scanning (micro-CT Quantum FX, Perkin Elmer^©^, Waltham, MA, USA) of the artificial stones and subsequent 3D segmentation using 3DSlicer software (NIH^©^) was used (Figure 2) [4,14,15].

### 2.4. Statistical Analysis

For the ablation volumes, a two-tail Student *t*-test was used with Rstudio and GraphPad Prism. *p* values of less than 0.05 were regarded as statistically significant.

## 3. Results

### 3.1. Optimal Displacement Velocity Experiments

**Figure 3:**
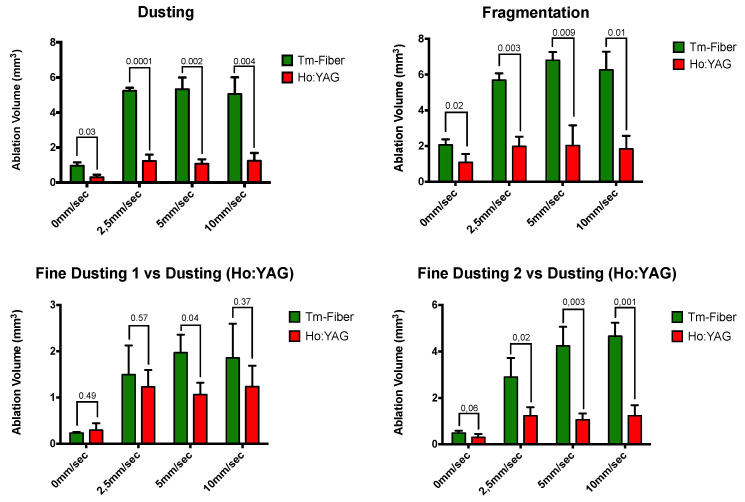
Comparative ablation volumes according to laser fiber displacement velocity.

Table 1 shows the results of the optimal displacement velocity experiments, reaching the maximal ablation volume found according to the laser fiber displacement velocity (LF-DV). For Ho:YAG lithotripsy, the ODV was higher in dusting compared to fragmentation mode (10 mm/s versus 5 mm/s, respectively). For Tm-Fiber, dusting and fragmentation OVDs were identical (5 mm/s). For Tm-Fiber, the ODV were 5 mm/s in dusting, fine dusting 1 and fragmentation but 10 mm/s in fine dusting 2. Tm-Fiber’s ODV was lower than Ho:YAG’s ODV in the dusting mode (5 mm/s versus 10 mm/s, respectively) but this difference was not consistent in the fragmentation mode (Table 2). The highest ODV (10 mm/s) was obtained in FD2 for Tm-Fiber and dusting for Ho:YAG (Figure 3).

### 3.2. Comparative Static and Dynamic Ablated Volumes

Table 2 shows the ablated volumes according to laser source, laser settings and LF-DV. Without displacement, ablation rates were at least two-fold higher with Tm-Fiber compared to Ho:YAG, except FD1 (Tm-Fiber vs. dusting (Ho:YAG). In dusting, Tm-Fiber was associated with a five-fold higher ablation volume at ODV (5.34 ± 0.66 vs. 1.24 ± 0.45, *p* = 0.001) (Table 2 and Table 3). In Fragmentation, a three-fold ratio was found (6.8 ± 0.45 vs. 2.02 ± 1.14, *p* = 0,009). Regarding the fine dusting modes, ablation volumes at ODV were 1.5-fold and four-fold higher in FD1 and FD2 compared to Ho:YAG in dusting (1.97 ± 0.39 vs. 1.24 ± 0.45, *p* = 0.18 and 4.66 ± 0.57 vs. 1.24 ± 0.45, *p* = 0.001, respectively). Regardless to the LF-DV, the ablation volumes with Tm-Fiber were significantly higher than Ho:YAG’s, except when comparing FD1 (0, 2.5 and 10 mm/s) and FD2 (0 mm/s) (Table 2, Figure 3).

## 4. Discussion

### 4.1. Optimal Displacement Velocity

#### 4.1.1. Difference between Tm-Fiber and Ho:YAG Lasers

This study firstly reports the optimal laser fiber displacement velocity for both Ho:YAG and Tm-Fiber lasers during urinary stone lithotripsy. We found that Tm-Fiber is associated with a lower ODV compared to Ho:YAG in dusting mode. It represents a safety parameter for Tm-Fiber use in clinical practice. Moreover, it may facilitate the laser “painting” at the surface of the stone during lithotripsy. Indeed, when using the “painting” technique, the operator aims to fire on a one locus-one laser pulse mode, which is as easy to perform as the laser fiber displacement velocity (LF-DV) is low. If the ODVs differ among laser parameters (lower with a higher pulse energy, Table 1) with the Ho:YAG laser, these findings were not consistent with the Tm-Fiber. Thus, the ODV was 5 mm/s in FD1, dusting and fragmentation. Our results could be explained by two distinct physical effects. Firstly, a “popcorn” effect could have occurred during our experiments at fine dusting 1 (0.05 J-300 Hz), with multiple laser pulses in the same location, increasing the distance between the stone phantom and the laser fiber. Consequently, this could have reduced the ablated volume [16]. It would have been interesting to compare the produced fragments’ size between Fine Dusting 1 and Fine Dusting 2 to support our explanation. Secondarily, a “burnback” effect could have been significant in fine dusting 2, with a greater degradation of the fiber tip, reducing its real output power [8,17]. Consequently, the ODV seems to depend more on the lithotripsy mode, defined by the pulse rate-pulse energy couple more than on the pulse rate only, at least for Tm-Fiber lasers as suggested recently [18]. More experiments are required to define the best regimens for each laser source: ODV, pulse rate and pulse energy.

Our study focused on the LF-DV on the stone surface, but other determinants of the laser stone ablation efficiency should be acknowledged. We have recognized the importance of the laser settings. As one of them, the pulse modulation (pulse duration and peak power (PP)) may have a major role. The longer pulse duration of Tm-Fiber, compared to Ho:YAG (even in long pulse setting) could require a lower LF-DV, in order to optimize the ablated volume [3,6]. As a potential consequence to the longer pulse duration, the morphology and the duration of the induced vapor channel created at the fiber tip could participate in the smaller ODV for Tm-Fiber [5]. If the mechanism of stone ablation associates photothermal and mechanical effects, the induced vapor bubbles take parts in both mechanisms [19,20,21]. A longer (duration and size) and wider bubble flow may be responsible for a greater ablated volume in a precise location, thereby reducing the ODV, according to our definition. Regarding the PP, a minimal level of 500 W is needed to obtain enough mechanical ablative effect. Ventimiglia et al. reported a 2000 to 20,000 W PP with Ho:YAG and rapid decrease of power during the laser pulse [21]. This high PP exposes to higher stone retropulsion and burnback effect, and lower ablation rates, explained by the initial overshoot occurring on the oscilloscopic profile. The Tm-Fiber, with its uniform staged oscilloscopic profile and lower PP (500 W max), but closer to the average power, requires a lower ODV at equal pulse rate–pulse energy settings.

#### 4.1.2. From In Vitro to Clinical Practice

The present study introduces a new concept in laser lithotripsy. If the laser-fiber displacement velocity may be instinctive to the majority of endourologists, no previous in vitro experiments intended to objectively define the ODV values among laser settings. We have emphasized the laser factors and laser fiber working distance that could have influenced the ablation efficiency, but other intra-operative factors must be noted. The surgeon’s technique and ability to dust stones necessarily has a major role in stone ablation, as the required efficacy (J/mm^3^) and efficiency (mm^3^/s) ratios are respectively lower and higher in expert centers compared to non-expert ones [7,9]. The stone composition could also influence ODV. In our experiments, we used hard synthetic stones, aiming to reproduce calcium oxalate monohydrate or calcium phosphate stones (representing fifty percent of the stone population) [22]. In vitro studies suggested that produced fragments in hard stones were wider than those with soft ones. Differently speaking, the morphology of the crater among stone composition could differ, and so ODV. As a result, we could suggest a lower ODV in soft stones (uric acid, calcium oxalate dihydrate and struvite). Furthermore, the production of significant fragments that would require basketting during flexible ureteroscopy is often correlated with the surgeon’s technique or experience or the laser parameters [4,6,7]. Consequently, operating at the ODV could help to minimize the risk of fragments’ production, and the time-consuming basketting duration (25% of the procedure) [7]. Finally, any surgeon has to displace the laser fiber at the stone surface according to the endoscopic vision and the produced fragments in order to avoid mucosa injuries. ODV could be individual, depending on its expertise and ability to dust.

### 4.2. Comparative Ablative Efficiency

The present study demonstrated that a static laser emission results in a lower ablated volume compared to dynamic laser emission, regardless the laser source, the laser settings or the LF-DV (Table 2). Our findings support the “painting technique” for dusting lithotripsy, consisting of a displacement at the surface of the stone to avoid several laser pulses in the same location. Despite the LF-DV, we report a 1.5 to 5-fold higher ablation volume with Tm-Fiber compared to Ho:YAG (Table 2). At same LF-DV, our study showed significantly higher ablation volume with Tm-Fiber, except in the FD (Tm-Fiber)-dusting (Ho:YAG) comparison. These findings are consistent with previous in vitro or in vivo studies [4,9,23]. At the ODV, only the FD1 lithotripsy mode (Tm-Fiber) is associated with a non-significant higher ablation volume, compared to the Ho:YAG laser in dusting mode (Table 3). This specific result can be explained by a low peak power in FD mode. Indeed, as mentioned before, a minimal level of PP is required to engage the stone breakage. As a limit of the first generation of Tm-Fiber, the used generator presents a 500 W PP in FD mode [21]. Our experiments confirms the in vitro superiority of Tm-Fiber over Low Power Ho:YAG laser for endocorporeal laser lithotripsy.

### 4.3. Strengths and Limitations

The present study is not devoid of limitations. Regarding the experimental setup, we have to discuss our definition of the ODV. We decided a priori that the higher ablation volume would be retained as the major criteria for ODV. We could have also used the characteristics of the ablated crater: deepness, width, length and produced fragments’ size. Consequently, even if our method has been validated by previous in vitro and in vivo studies, we could have analyzed the stone samples using a profilometric method [4,7,9,15,24]. A further dedicated experiment should directly compare both profilometric and three-dimensional segmentation methods. Moreover, we must acknowledge a major limitation of our work: the low rate of studied LF-DV. We used a robotic-arm, executing small displacements but in a narrow range of velocity, in order to preserve the precision of the displacement. In dusting (Ho:YAG) and FD1 (Tm-Fiber), we may not have presented the best ODV as we cannot be certain to have reached the highest ablation volume. Enlarging the LF displacement distance could respond to this limitation, but the LF degradation would be greater and the contact mode, i.e., LF in contact with the stone, consequently altered. Furthermore, there was a compromise for analysis between the LF dynamic characteristics and the stone sample. Moreover, we have only presented the results with one LF core-diameter. To our knowledge, there is no available data with identical or different LF core-diameter. Smaller LFs (down to 50 µm) could result in lower ODV at same irradiance (W/cm^2^) or energy density (J/cm^2^), acknowledging the induced vapor bubbles would be smaller and shorter [5]. Regarding the pulse modulation, we must recognize we used a previous version of Tm-Fiber, without modulation of the peak-power. In FD, the peak-power was consequently lower than in dusting and fragmentation modes. This could have altered our findings and lowered the ODV, especially for low pulse energies–high pulse rates (fine dusting 1 and 2). Furthermore, our Ho:YAG laser was a low power (LP) generator (30 W Maw Power), without Moses’ Technology^©^ [25]. If high power (HP) Ho:YAG laser (up to 120 W) have been suggested to improve stone ablation, compared to low power ones, their interest is still debated in ELL [17,26,27]. A recent meta-analysis reported similar outcomes between LP and HP-Ho:YAG generators [28]. Therefore, we can analyze our results only for LP-Ho:YAG and Tm-Fiber.

Despite these limitations, we firstly introduce the concept of optimal displacement velocity in ELL. More in vitro and clinical studies are needed to define the exact ODV beyond laser source and settings.

## 5. Conclusions

The present in vitro study introduces the concept of optimal displacement velocity during endocorporeal laser lithotripsy. Tm-Fiber is associated with a lower ODV than low power Ho:YAG laser, facilitating the “painting” dusting technique. ODV may rely on several parameters, including a surgeon’s ability to dust, that is not yet defined. When the laser fiber is moving at the surface of the stone, ablation volumes are higher.

## Figures and Tables

**Table 1:** Optimal displacement velocity according to laser setting and laser source.

Laser Source	Lithotripsy Mode	Optimal Displacement Velocity (mm/s, Ablated Volume (mm^3^))
Tm-Fiber	Fine Dusting 1 (0.05 J-300 Hz)	5 mm/s (1.97 mm^3^)
	Fine Dusting 2 (0.15 J-100 Hz)	10 mm/s (4.66 mm^3^)
	Dusting (0.5 J-30 Hz)	5 mm/s (5.34 mm^3^)
	Fragmentation (1 J-15 Hz)	5 mm/s (6.8 mm^3^)
Ho:YAG	Dusting (0.5 J-30 Hz)	5 mm/s (1.24 mm^3^)
	Fragmentation (1 J-15 Hz)	10 mm/s (2.02 mm^3^)

**Table 2:** Ablation volumes according to laser fiber displacement velocity, laser settings and laser source.

Lithotripsy Mode	Laser Fiber Displacement Velocity (mm/s)	Ablation Volume (mm^3^)		
		Tm-Fiber	Ho:YAG	*p*-Value
Dusting	0	0.96 ± 0.19	0.3 ± 0.15	**0.003**
	2.5	5.24 ± 0.17	1.23 ± 0.36	**0.0001**
	5	**5.34 ± 0.66**	1.07 ± 0.26	**0.002**
	10	5.05 ± 0.95	**1.24 ± 0.45**	**0.004**
Fragmentation	0	2.06 ± 0.31	1.09 ± 0.46	**0.02**
	2.5	5.69 ± 0.38	1.98 ± 0.54	**0.003**
	5	**6.8 ± 0.45**	**2.02 ± 1.14**	**0.009**
	10	6.27 ± 1	1.84 ± 0.72	**0.01**
**Lithotripsy Mode**	**Laser Fiber Displacement Velocity (mm/s)**	**Ablation Volume (mm^3^)**		
		**Tm-Fiber** **(0.05 J-300 Hz)**	**Ho:YAG (0.5 J-20 Hz)**	***p*-Value**
Fine Dusting 1 (Tm-Fiber) vs. Dusting (Ho:YAG)	0	0.24 ± 0.02	0.3 ± 0.15	0.49
	2.5	1.49 ± 0.63	1.23 ± 0.36	0.57
	5	**1.97 ± 0.39**	1.07 ± 0.26	**0.04**
	10	1.86 ± 0.74	**1.24 ± 0.45**	0.37
**Lithotripsy Mode**	**Laser Fiber Displacement Velocity (mm/s)**	**Ablation Volume (mm^3^)**		
		**Tm-Fiber (0.15 J-100 Hz)**	**Ho:YAG (0.5 J-20 Hz)**	***p*-Value**
Fine Dusting 2 (Tm-Fiber) vs. Dusting (Ho:YAG)	0	0.49 ± 0.09	0.3 ± 0.15	0.06
	2.5	2.9 ± 0.82	1.23 ± 0.36	**0.02**
	5	4.24 ± 0.82	1.07 ± 0.26	**0.003**
	10	**4.66 ± 0.57**	**1.24 ± 0.45**	**0.001**

**Table 3:** Comparative ablation volumes at optimal displacement velocity.

Lithotripsy Mode	Ablation Volume (mm^3^)		
	Tm-Fiber	Ho:YAG	*p*-Value
Fine Dusting 1 (Tm-Fiber) vs. Dusting (Ho:YAG)	1.97 ± 0.39	1.24 ± 0.45	0.18
Fine dusting 2 (Tm-Fiber) vs. Dusting (Ho:YAG)	4.66 ± 0.57	1.24 ± 0.45	**0.001**
Dusting	5.34 ± 0.66	1.24 ± 0.45	**0.001**
Fragmentation	6.8 ± 0.45	2.02 ± 1.14	**0.009**

## Data Availability

Data are available by contacting authors.
